# Dosage of antipsychotics in China routine practice

**DOI:** 10.1192/j.eurpsy.2021.980

**Published:** 2021-08-13

**Authors:** T. Zhang, R. Chi, T. Wu, Y. Xu, W. Dong

**Affiliations:** 1 Epidemiology, Janssen Research and Development, Beijing, China; 2 Peking University The Sixth Hospital, Institute Of Mental Health; National Clinical Research Center For Mental Health Disorders & Key Laboratory Of Mental Health, Ministry Of Health, Peking University, Peking University the Sixth Hospital, Beijing, China

**Keywords:** DDD, dosage, antipsychotic, real-world database

## Abstract

**Introduction:**

The antipsychotic dosage of Chinese schizophrenia patients has rarely been studied, although nonstandard dosage has impact on prognosis.

**Objectives:**

To describe the dosage of antipsychotics in China routine practice.

**Methods:**

This was a retrospective cohort study using de-identified data from a Chinese mental health hospital. The included patients were adults (≥18 years) with at least one diagnosis of schizophrenia (ICD-10: F20) and one prescription of any antipsychotic between 2014 and 2019. Date of first identified antipsychotic prescription was defined as index date, patients were followed up until last prescription of antipsychotics, end of 2019, or discontinuation (>60 days without antipsychotic prescription), whichever was earliest. Dosage was summarized using defined daily dose (DDD), calculated by cumulative average daily dose (CAD) with a unit of DDDs/day, i.e., total DDDs of all antipsychotics in follow-up period divided by total days of follow-up. CAD was categorized into low (<0.5 DDDs/day), moderate (0.5-1.5 DDDs/day), and high (>1.5 DDDs/day) groups.

**Results:**

13554 patients were included with an average follow-up of 269.9 days. Median CAD was 0.8 DDDs/day (IQR=0.5-1.3), patients with hospitalization during follow-up and used multiple antipsychotics at the same time had larger median CAD, 1.0 DDDs/day and 1.2 DDDs/days, respectively. There were 3245 (23.9%), 7627 (56.3%), and 2682 (19.8%) patients in low, moderate, and high groups, respectively. The median CAD of high dosage group was 2.5 DDDs/day (IQR=1.9-10.5).

**Conclusions:**

CAD of most Chinese schizophrenia patients was low or moderate. Association between CAD and hospitalization and multiple concurrent antipsychotics merit further research.

